# Semantic‐specific and domain‐general mechanisms for integration and update of contextual information

**DOI:** 10.1002/hbm.26454

**Published:** 2023-10-03

**Authors:** Francesca M. Branzi, Matthew A. Lambon Ralph

**Affiliations:** ^1^ Department of Psychological Sciences Institute of Population Health, University of Liverpool Liverpool UK; ^2^ MRC Cognition & Brain Sciences Unit The University of Cambridge Cambridge UK

**Keywords:** context, domain‐general, fMRI, multiple demand‐network, semantic control, language network

## Abstract

Recent research has highlighted the importance of domain‐general processes and brain regions for language and semantic cognition. Yet, this has been mainly observed in executively demanding tasks, leaving open the question of the contribution of domain‐general processes to natural language and semantic cognition. Using fMRI, we investigated whether neural processes reflecting context integration and context update—two key aspects of naturalistic language and semantic processing—are domain‐specific versus domain‐general. Thus, we compared neural responses during the integration of contextual information across semantic and non‐semantic tasks. Whole‐brain results revealed both shared (left posterior‐dorsal inferior frontal gyrus, left posterior inferior temporal gyrus, and left dorsal angular gyrus/intraparietal sulcus) and distinct (left anterior‐ventral inferior frontal gyrus, left anterior ventral angular gyrus, left posterior middle temporal gyrus for semantic control only) regions involved in context integration and update. Furthermore, data‐driven functional connectivity analysis clustered domain‐specific versus domain‐general brain regions into distinct but interacting functional neural networks. These results provide a first characterisation of the neural processes required for context‐dependent integration during language processing along the domain‐specificity dimension, and at the same time, they bring new insights into the role of left posterior lateral temporal cortex and left angular gyrus for semantic cognition.

## Graphical abstract

Semantic control and domain‐general control are partially dissociable. IFG, pLTC, and AG revealed both domain‐specific and domain‐general subregions. Domain‐specific and domain‐general networks work in *tandem* for context integration.

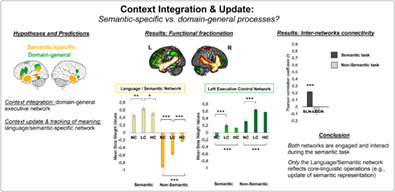

## 1 INTRODUCTION

Understanding the news reports on the radio or sequential paragraphs while reading a book relies on the ability of the semantic system to integrate information over time to build meaningful representations. Whilst semantic integration is often error‐free and apparently effortless, the cognitive challenges are non‐trivial. The sensory inputs to our semantic system are constantly changing and, therefore, the semantic system is required over an extended period to integrate semantic information to build a semantic representation from the torrent of words and nonverbal stimuli. This representation, that here and previously (see Branzi, Humphreys, et al., 2020) we called a “semantic gestalt,” is continuously evolving in a context‐dependent fashion: As each unit of semantic information is integrated, the representation is revised and sometimes fully updated when the context changes abruptly (see also the “sentence gestalt model,” McClelland et al., 1989; St John & McClelland, 1990; Rabovsky et al., 2018).

Recently, we established that the integration of semantic information into a coherent contextual representation (or semantic gestalt) relies on the workings of two neural systems: the first is crucial for the formation of a semantic gestalt (time‐extended integration/formation of a semantic representation), whilst the second is important for buffering information into a schema representation (Branzi, Humphreys, et al., 2020; Branzi, Pobric, et al., 2021).

Our previous research, however, left important issues unaddressed. For instance, it is unclear when and if the neural mechanisms that support buffering of information and its integration into a contextual representation reflect domain‐specific or domain‐general combinatorial operations. Furthermore, we still do not know very much about the semantic control processes required during the formation of contextually integrated representations. For instance, are the semantic control demands required during integration of partially incoherent contexts (*hard* semantic integration) analogous to domain‐general executive control processes? And, to what degree are the neural mechanisms, underpinning semantic control during context integration, specialised for this domain? The present using functional magnetic resonance imaging (fMRI) study was designed to address these important questions.

Semantic cognition is supported by a distributed neural network of frontoparietal and temporal regions (Branzi, Martin, et al., 2020; Branzi, Martin, & Paz‐Alonso, 2021; Canini et al., 2016; Jefferies, 2013; Jefferies & Lambon Ralph, 2006; Lambon Ralph et al., 2017). Within this network, some brain regions have a key role in manipulating activation within the semantic representational system to generate semantic behaviours that are appropriate for the context in which they occur (Jackson, 2021; Jackson et al., 2021; Lambon Ralph et al., 2017; Noonan et al., 2013). These brain regions include the inferior frontal gyrus (IFG) and the posterior lateral temporal cortex (pLTC) (particularly the posterior middle temporal gyrus [pMTG]), which are strongly interlinked with functional and anatomical connections (Catani et al., 2005; Jung et al., 2017; Saur et al., 2008; Turken & Dronkers, 2011) and often co‐activated for flexible and controlled retrieval of context or task‐relevant semantic information (Hodgson et al., 2021; Jefferies & Lambon Ralph, 2006; Noonan et al., 2013; Whitney et al., 2012; Whitney, Kirk, et al., 2011). Accordingly, we revealed that a discordant change in the semantic context or topic during naturalistic language processing induces strong activations in both IFG and pLTC/pMTG (Branzi, Humphreys, et al., 2020).

The left ventral angular gyrus (AG) is another key region for semantic cognition (Jackson, 2021; Lambon Ralph et al., 2017). The left ventral AG has been associated with a domain‐general and functionally‐graded multimodal buffer (Humphreys et al., 2020; Humphreys et al., 2021; Humphreys & Lambon Ralph, 2015). The peak location for buffering information seems to shift across the ventral AG, depending on the variations in long‐range connections, such that anterior ventral AG is maximally recruited for buffering verbal/auditory information, core mid‐AG for episodic information, and posterior ventral AG for visuospatial content (Humphreys et al., 2020). Consistent with this view, we demonstrated that ‘disrupting’ the left core‐mid AG's activity during buffering of contextual information impairs the encoding of contextually integrated representations (Branzi, Pobric, et al., 2021).

The left dorsal AG/intraparietal sulcus (dorsal AG/IPS) has been also associated with semantic cognition (Humphreys et al., 2021; Lambon Ralph et al., 2017; Noonan et al., 2013). However, unlike the ventral AG, the dorsal AG/IPS is strongly connected to the “multiple demand network,” a set of brain regions activated across a broad range of executively demanding tasks (Assem et al., 2020; Duncan, 2010; Duncan et al., 2020; Jung et al., 2022). Accordingly, we showed that dorsal AG/IPS is strongly recruited during the integration of partially incoherent contexts (*hard* semantic integration) (Branzi, Humphreys, et al., 2020; Branzi, Pobric, et al., 2021).

All regions noted above are potentially important for semantic cognition. Yet, whether the regions implicated in the integration of coherent and incoherent contexts do so through domain‐specific or domain‐general processes remains unclear. Indeed, specific sub‐regions of left IFG left pLTC and left AG might support domain‐general control processes (Hodgson et al., 2021). These claims are mainly motivated by the observation of some overlap between the neural network for semantic control and the multiple demand network (Geranmayeh et al., 2014; Hodgson et al., 2021; Humphreys & Lambon Ralph, 2015). However, only a minority of studies have compared semantic versus non‐semantic tasks tapping similar cognitive operations (e.g., context integration), and those that have, obtained mixed results (Humphreys et al., 2020; Humphreys & Lambon Ralph, 2015; Humphreys & Lambon Ralph, 2017; Quillen et al., 2021). Perhaps more problematic, the evidence of shared neural substrates between semantic and domain‐general control processes comes from paradigms where typically little or no context information is provided during semantic processing, and/or where the main task is accompanied by a secondary task (Hodgson et al., 2021; Humphreys & Lambon Ralph, 2015). As a result, such paradigms may recruit domain‐general control processes and the multiple demand network, which is highly sensitive to task demands. However, this recruitment does not necessarily speak to the role of this network in core semantic operations, such as for instance formation and updating of a continuously evolving representation, that is, the target of the present study.

**FIGURE 1:**
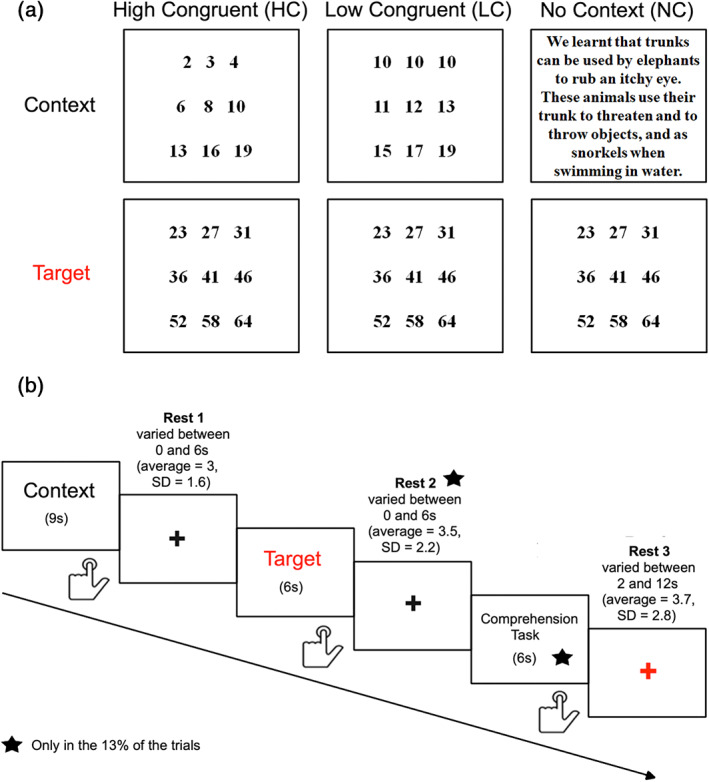
(a) Example of trials in the non‐semantic task, including HC, LC, and NC conditions. (b) Schematic depiction of trial structure and timing for both semantic and non‐semantic tasks.

To fully characterise the role of left IFG, left pLTC, and left AG for context integration and update we conducted an fMRI study where we compared semantic versus non‐semantic time‐extended receptive tasks, made up of either two paragraphs of written text or number sequences, respectively (see Figure 1 and Branzi, Humphreys, et al., 2020; see Tables S1 and S3). In both tasks, there were three variations in respect to the relationship between the two paragraphs: (a) high congruency (HC condition)—the second paragraph continued with the same type of information, that is, a coherent continuation of the same meaning/number sequence; (b) low congruency—where the second paragraph represented a change in the meaning or number sequence (LC condition); or (c) no context (NC condition)—the second paragraph was not preceded by any informative context (a different kind of material altogether). The comparison of HC and LC against NC condition allowed us to establish the brain regions important for integration of contextual information, that is, how the brain builds an evolving representation. Instead, the comparison of LC against HC conditions allowed us to identify the brain regions involved in updating contextual information during integration. To address the key question of this study, for both contrasts, we examined the extent to which similar or dissimilar brain regions were engaged in semantic and non‐semantic tasks.

We expected both left IFG and pLTC to be implicated in the integration of contextual information (LC and HC > NC), especially when integration demands increase (LC > HC) (Jackson, 2021; Noonan et al., 2013). However, we expected the recruitment of different subregions depending on the type of stimuli/task (Hodgson et al., 2021; Humphreys & Lambon Ralph, 2017). Specifically, for left IFG, we expected anterior‐ventral portions (~Pars Orbitalis) to show domain‐specific effects, that is, to be positively engaged only in the semantic task (Hodgson et al., 2021). On the other hand, we expected more posterior‐dorsal portions of left IFG (~Pars Triangularis and Pars Opercularis) to show domain‐general effects (Badre & Wagner, 2002; Branzi, Martin, et al., 2020; Dobbins & Wagner, 2005).

For left pLTC, we expected left pMTG to be exclusively involved in semantic control (Hodgson et al., 2021; Humphreys & Lambon Ralph, 2017), whilst the inferior portion (posterior inferior temporal gyrus [pITG]) to show domain‐general responses, in accord with evidence showing a strong link between neural activity in this region, the multiple demand network and domain‐general control processes (Assem et al., 2020; Hodgson et al., 2021).

We expected left AG to be involved in context integration (HC and LC > NC) (Branzi, Pobric, et al., 2021). However, recent explorations have shown that the AG is not a homogenous region, with some sub‐regions implicated in domain‐specific and others in domain‐general processes. Specifically, dorsal AG/IPS is active across a large variety of tasks and cognitive activities, especially when task or condition demands increase (Humphreys & Lambon Ralph, 2015; Humphreys & Lambon Ralph, 2017; Noonan et al., 2013). Accordingly, we expected left dorsal AG/IPS to be engaged in both tasks during integration of contextual information, and more strongly for LC as compared to HC conditions (Noonan et al., 2013). In contrast, the left anterior ventral AG shows a preference for verbal stimuli, such as sentences or narratives, especially when task demands decrease (Branzi, Humphreys, et al., 2020; Humphreys & Lambon Ralph, 2015). However, the profile of activation/deactivation of the ventral AG in this study is hard to predict from the current literature. In fact, on the one hand, some results have shown that the ventral AG is positively engaged for the processing of verbal/semantic stimuli, but deactivated for numerical stimuli (Branzi, Humphreys, et al., 2020). On the other hand, other results suggest that this region might be positively engaged during numerical and verbal/semantic processing alike, especially when control demands decrease (Humphreys & Lambon Ralph, 2015).

To fully characterise the profile of engagement of different IFG, pLTC, and AG subregions during semantic and non‐semantic tasks, we also employed multivariate functional connectivity analysis, specifically Independent Component Analysis (ICA). This data‐driven approach allowed us to (1) measure if the above‐mentioned brain regions exhibited a “yoked” activation, that is, if they formed coherent functional networks; (2) establish if the networks that support contextual integration and update show domain‐specific or domain‐general patterns of engagement; and finally, (3) examine the extent to which interactions between domain‐specific and domain‐general networks are modulated by the task/stimuli domain.

Based on previous evidence (Geranmayeh et al., 2014; Humphreys et al., 2020), we expected a frontoparietal network including domain‐general brain regions (i.e., posterior‐dorsal portions of IFG, left dorsal AG/IPS, and pLTC/pITG) to be similarly involved in the two tasks, and to reflect context integration processes (HC and LC > NC). Instead, we expected domain‐specific networks to include domain‐specific subregions, for example, the anterior temporal lobe for the semantic task. Since the language/semantic network—but not the multiple demand network—may track semantic content specifically (Diachek et al., 2020), we expected domain‐specific networks specifically to support context updates (LC > HC). Finally, we expected some spatial overlap and interaction between the domain‐general network for context integration and domain‐specific networks for context update. Specifically, we expected spatial overlap in brain regions reflecting domain‐general neural responses and inter‐networks functional interactions to be modulated by the type of task or stimuli employed.

## 2 MATERIALS AND METHODS

### 2.1 Participants

All participants were native English speakers, undergraduate and postgraduate students, with no history of neurological or psychiatric disorders, and normal or corrected‐to‐normal vision. Twenty‐four volunteers took part in the semantic task (average age = 23 years, standard deviation (SD) = 3; *N* female = 19; age range = 19–30 years) (Branzi, Humphreys, et al., 2020). As a result of technical issues during the scanning session, only data from 22 participants (average age = 23 years, SD = 3; *N* female = 17; age range = 19–30 years) were usable for fMRI data analyses. Twenty‐four volunteers took part in the non‐semantic task (average age = 22 years, SD = 2; *N* female = 19; age range = 19–29 years). Both experiments were approved by the local ethics committee. Written informed consent was obtained from all participants.

### 2.2 Stimuli

#### 2.2.1 Semantic task

A total of 40 narrative pairs were created. For each narrative pair, the same second paragraph (target) was preceded by different first paragraphs (contexts) that could be either high‐congruent (i.e., HC) or low‐congruent (i.e., LC) with the target in terms of meaning. Both HC and LC context paragraphs could be integrated with the final target paragraphs, though a reworking of the evolving semantic context was required after LC contexts only, because of a shift in the semantic context (see Table S1 for a comprehensive list of the stimuli). Homonym words (e.g., bank) were employed near the initial part of the target paragraph to determine a shift in the semantic context for LC conditions.

To confirm that HC and LC conditions differed in the semantic associative strength between contexts and targets, we quantified semantic relatedness between the contexts and targets, for both HC and LC conditions, in multiple ways. First, we employed latent semantic analysis (LSA) (Landauer & Dumais, 1997) to measure the semantic relationship between words based on the degree to which they are used in similar linguistic contexts. Thus, for each narrative pair, context and target paragraphs were converted into vectors of words that were successively compared, using a cosine similarity metric. From this comparison, an LSA value reflecting the associative strength between the context and target was obtained for both conditions. Results from LSA confirmed that semantic associative strength between the (same) target and the context was higher for HC (average score = 0.41, SD = 0.16) than LC conditions (average score = 0.23, SD = 0.1) [*t* (78) = 5.996, *p* < .001].

Second, as reported previously (Branzi, Humphreys, et al., 2020), we asked a group of independent participants to rate how semantically related contexts and targets were (0–5 scale). The results of this pre‐experimental rating indicated that HC (average score = 4.4, SD = 0.4) and LC (average score = 2.3, SD = 0.4) conditions were different [*t* (9) = 10.626, *p* < .001]. Moreover, to ensure that participants could perceive the shift of semantic context during the study, at end of each narrative the question “Was there any change of semantic context between part 1 and part 2?” was posed. Only pairs of narratives on which at least the 90% of participants responded correctly to the questions were employed in the study.

Finally, another condition was included in the experimental design to measure the context integration processes. Specifically, in the no‐context (NC) condition, the target (the same as in HC and LC conditions) was preceded by a string of numbers that could include from one to four‐digit numbers.

#### 2.2.2 Non‐semantic task

To compare the neural basis of context integration across semantic and non‐semantic tasks, response times (RTs) were matched across the semantic and non‐semantic domains. This was achieved by conducting a pilot study with 10 participants on preliminary versions of the non‐semantic experiment outside the scanner. Based on these participants' behavioural data, we iteratively adjusted several aspects of the stimuli of the non‐semantic task, until we obtained a final version in which conditions were matched to the semantic task in terms of RTs (Branzi, Humphreys, et al., 2020). Like the semantic task, a total of 40 stimuli pairs, each one composed by two “paragraphs,” were created. However, the content of each paragraph corresponded to numerical sequences. Thus, each paragraph contained a numerical sequence presented over three rows, with three numerical elements per row (see Figure 1a and Table S3 for a comprehensive list of the stimuli). For each pair, the same second paragraph (target) was preceded by different first numerical paragraphs (contexts) that could be either high‐congruent (i.e., HC) or low‐congruent (i.e., LC) with the target paragraph's numerical sequence. Both HC and LC context paragraphs could be associated with the target's, though a change in the context was required after LC contexts only, because of a shift in the numerical sequence (Figure 1a). In fact, a specific number presented near the start (first or second number) of each target condition represented the exact point in the paragraph where the shift of numerical rule should have been experienced for LC conditions (see Table S3). Finally, as per the semantic task, another condition was included in the design to measure the context integration processes. Specifically, in the NC condition, the target paragraph (the same as that used in the HC and LC conditions) was preceded by a verbal paragraph taken from the semantic task (Figure 1a).

### 2.3 Task procedures

#### 2.3.1 Semantic task

There were 40 items per condition presented using an event‐related design with the most efficient ordering of events determined using Optseq (http://www.freesurfer.net/optseq). The details of the trial presentation are reported in Figure 1b. Time was intermixed between trials and varied between 2 and 12 s (average = 3.7, SD = 2.8). During this time a red fixation cross was presented. The red colour was used in order to mark the end of each trial (each trial composed by a context and a target). A black fixation cross was presented between contexts and targets and its duration varied between 0 and 6 s (average = 3, SD = 1.6). Each context paragraph was presented for 9 s, followed by the target paragraph, which was displayed for 6 s.

Participants were asked to read silently both contexts and targets. Our volunteers were instructed to press a button when reaching the end of each paragraph (for both contexts and targets). The instruction emphasised speed, but also the need to understand the meaning of contexts and targets, since at the end of some of the trials, participants would be asked some questions about the content of the stimuli. We specified that, in order to perform this task, it would be necessary to process contexts and targets together. Hence, following 13% of the trials, a comprehension task was presented to ensure that participants were engaged in the task (see Table S2). When this happened, the target item was followed by a question displayed on the screen for 6 s, and participants were asked to provide a response (true/false) via button press. A fixation cross, between the target and the question, was presented for a variable duration between 0 and 6 s (average = 3.5, SD = 2.2). Before starting the experimental study, all participants signed an informed consent and were given written instructions. Then, they completed a practice session to familiarise themselves with the task. The stimuli used in the practice session were different from those used in the experimental study.

#### 2.3.2 Non‐semantic task

There were 40 items per condition presented with the same procedure as in the semantic task (see above and Figure 1b). Thus, participants were asked to read silently both contexts and targets (displayed on the screen for 9 and 6 s, respectively), process them as a unique numerical sequence, and press a button when reaching the end of each paragraph (for both contexts and targets). However, to ensure that our volunteers were engaged in similar processing as in the semantic task (i.e., the same item‐by‐item and sentence‐by‐sentence integration processes), we also instructed participants to detect the combinatorial rule linking the first three numbers in the first row (e.g., incremental addition, see Figure 1a) with the content of the other remaining rows.

Furthermore, we informed participants that sometimes the combinatorial rule would change, but that their task would nevertheless be the same, that is, read the numbers presented in the context and target paragraphs silently, and detect the new combinatorial rule. The instruction emphasised speed, but also the need to process the information included in contexts and targets since at the end of some trials participants would be asked questions on the content of the stimuli. As per the semantic task, following 13% of the trials, a comprehension task was presented to ensure that participants were engaged in the task (see Figure 1b). When this happened, the target item was followed by a question displayed on the screen for 6 s, and participants were asked to provide a response (true/false) via button press. The questions pertained to the combinatorial rule(s) linking the number sequences, as well as the specific numbers presented in the context and target paragraphs (see Table S4). A fixation cross between the target and the question was presented for a variable duration between 0 and 6 s (average = 3.5, SD = 2.2). Before starting the experimental study, all participants signed an informed consent and were given written instructions. Then, they completed a practice session to familiarise themselves with the task. The stimuli used in the practice session were different from those used in the experimental study.

### 2.4 Task acquisition parameters

Images for both semantic and non‐semantic tasks were acquired using a 3T Philips Achieva scanner using a dual gradient‐echo sequence, which is known to have improved signal relative to conventional techniques, especially in areas associated with signal loss (Halai et al., 2014). Thus, 31 axial slices were collected using a time to repetition (TR) = 2 s, short and long time to echo (TE) = 12 and 35 ms, flip angle = 95°, 80 × 79 matrix, with resolution 3 × 3 mm, slice thickness 4 mm. For each participant, 1492 volumes were acquired in total, collected in four runs of 746 s each.

### 2.5 Data analysis

#### 2.5.1 Behavioural data analyses

Since both tasks were reading tasks, accuracy measures could not be recorded. Instead, speed of reading (RTs) was analysed via a repeated‐measures analysis of variance (ANOVA), with “Condition” as a factor with three levels (NC, LC, and HC conditions) and “Task” as a factor with two levels (semantic task and non‐semantic task). To examine whether participants were similarly engaged in both tasks, we also computed an ANOVA including the percentage of given responses, with “Condition” as a factor with three levels (NC, LC, and HC conditions) and “Task” as a factor with two levels (semantic task and non‐semantic task). Bonferroni correction for multiple comparisons was applied to assess significant effects. Bonferroni‐corrected *p*‐values were reported accordingly. Finally, correction for non‐sphericity (Greenhouse–Geisser procedure) was applied to the degrees of freedom and *p*‐values associated with factors having more than two levels (i.e., Condition).

#### 2.5.2 fMRI data analyses

##### Preprocessing

The dual‐echo images were averaged. Data were analysed using SPM12. After motion correction, images were co‐registered to the participant's T1 image. Spatial normalisation into Montreal Neurological Institute (MNI) space was computed using DARTEL (Ashburner, 2007), and the functional images were resampled to a 3 × 3 × 3 mm voxel size and smoothed with an 8 mm FWHM Gaussian kernel.

#### 2.5.3 First‐level analysis

##### General linear modelling (GLM)

The data were filtered using a high‐pass filter with a cut‐off of 128 s. For both datasets, we ran a GLM model in which, at the individual subject level, each condition of interest was modelled with a separate regressor (target NC, target LC, and target HC) with time derivatives added, and events were convolved with the canonical hemodynamic response function, starting from the onset of the target paragraph. The context paragraphs (NC, LC, and HC contexts) and the comprehension task were modelled as regressors. However, these data were not further analysed because they were not relevant for the scope of the present study. Each condition was modelled as a single event with a duration corresponding to 6 s (target conditions and comprehension task trials) or 9 s (context conditions). Motion parameters were entered into the model as covariates of no interest.

#### 2.5.4 Second‐level analysis

##### Context integration and update in semantic and non‐semantic tasks

To establish which brain regions were similarly engaged during contextual integration (HC and LC > NC) and update (LC > HC) of semantic versus non‐semantic information, as a first step, we conducted a flexible factorial 2 (semantic task, non‐semantic task) × 3 (HC, LC, NC target condition) ANOVA, where subjects were treated as a random effect. The factor matrix included images derived from the first‐level analysis, relative to the NC, LC, and HC target regressors from both semantic and non‐semantic tasks. The main effects and interactions relative to the effects of interest were examined via whole‐brain analyses (*F*‐tests). These statistical maps were thresholded at *p* < .001 for voxel intensity, and *p* < .05 (family‐wise error (FWE)‐correction for multiple comparisons) for clusters. We also conducted a conjunction analysis to reveal which voxels showed, for each specified contrast of interest (i.e., HC and LC > NC and LC > HC), significant effects in both tasks. As a second step for this analysis, we investigated the direction of any potential difference between semantic and non‐semantic tasks and conditions, as well as the engagement of these regions as compared to rest. To this end, the contrast estimates were extracted and plotted via region of interest (ROI) analysis using spheres of 10 mm radius. The specific MNI coordinates for the ROI analysis, however, were derived from a recent large‐scale meta‐analysis study on the neural basis of semantic processing (Jackson, 2021, see Table 1). The data from ROI analysis were then analysed via ANOVAs, where “Task” was a factor with two levels (semantic and non‐semantic), and “Condition” was a factor with three levels (NC, LC, and HC targets). Bonferroni correction for multiple comparisons was applied to assess significant effects. Bonferroni‐corrected *p*‐values were reported accordingly. Finally, correction for non‐sphericity (Greenhouse–Geisser procedure) was applied to the degrees of freedom and *p*‐values associated with factors having more than two levels (i.e., Condition). For completeness, we also report the following post‐hoc analyses: (1) whole‐brain results for context integration (HC and LC > NC) and update (LC > HC) in semantic and non‐semantic tasks, separately; (2) whole‐brain results for semantic > non‐semantic tasks (and vice versa) for both context integration (HC and LC > NC) and update (LC > HC) (see Figures S1 **–** S3).

#### 2.5.5 Task group spatial ICA in semantic and non‐semantic tasks

Spatial ICA applied to fMRI data identifies temporally coherent networks by estimating maximally independent spatial sources, referred to as spatial maps, from their linearly mixed fMRI signals, referred to as time courses (Calhoun et al., 2001). We employed ICA to examine whether key brain areas revealed by univariate analyses were coupled with similar brain regions across semantic and non‐semantic tasks to support context integration and update. Furthermore, with ICA we aimed to reveal whether functional interactions between domain‐specific and domain‐general networks were modulated by the type of stimuli (see Introduction).

The pre‐processed fMRI data of both semantic and non‐semantic tasks were analysed together in a group spatial ICA using the GIFT toolbox (http://mialab.mrn.org/software/gift) (Calhoun et al., 2001) to decompose the data into components. GIFT was used to concatenate the subjects' data, and reduce the aggregated data set to the estimated number of dimensions using principal component analysis, followed by ICA using the Infomax algorithm (Bell & Sejnowski, 1995). Subject‐specific spatial maps and time courses were estimated using GICA back‐reconstruction method based on principal component analysis compression and projection (Calhoun et al., 2001).

The number of independent components estimated within the data was 33. The estimation was achieved by using the Minimum Description Length criteria, first per each individual dataset and then computing the group mean. The obtained 33 independent components were inspected in order to exclude from the analysis artefactual and noise‐related components. Similar to previous studies (Geranmayeh et al., 2014; Griffanti et al., 2017), the criterion for assigning components as artefacts was based on the spatial maps attained as a result of the one sample *t*‐tests (threshold for voxel‐wise significance was set at *p* < .05, corrected for FWE). The spatial maps were visually compared with the SPM grey matter template. Only components that had the majority of activity within the grey matter were selected (*N* = 26).

#### 2.5.6 Establishing task‐related functional networks in semantic and non‐semantic tasks

The 26 independent components were labelled according to the resting state networks template provided in the GIFT toolbox. Then, a multiple regression analysis (implemented as a “temporal sorting” function in GIFT) between independent component's and task model's time courses for each participant was conducted. This analysis allowed identifying which independent components reflected task‐related functional networks in both tasks. In detail, for each participant, the design matrix used for the GLM analysis, where rest periods were modelled implicitly as task baseline, was employed. For each independent component, the multiple regression analysis generated three beta weight values (one for each condition: NC, LC, and HC) that were averaged across runs and participants. Beta weight values represent the correlations between the time courses of the independent components and the canonical hemodynamic response model for each task condition. These values are thought to reflect the engagement of the functional networks during specific task conditions (e.g., Xu et al., 2013).

After extracting the beta weight values for each independent component associated with each task condition in each task, task‐relatedness for each component was assessed. This was achieved by testing group means of averaged beta weight values for each task condition against zero (one‐sample *t*‐tests, *p* < .05). Hence, a positive/negative beta weight value, significantly different from zero, indicated an increase/decrease in activity of the independent component during a specific task condition relative to the baseline condition (i.e., rest). Having established the task‐related functional networks, a repeated‐measures ANOVA was used to assess the main differences between beta weight values across different task conditions and tasks, for each network. Bonferroni correction for multiple comparisons was applied to assess significant effects. Bonferroni‐corrected *p*‐values were reported accordingly. Finally, correction for non‐sphericity (Greenhouse–Geisser procedure) was applied to the degrees of freedom and *p*‐values associated with factors having more than two levels (i.e., Condition).

#### 2.5.7 Functional network connectivity (FNC) analysis

To explore functional interactions between neural networks of interest (domain‐specific and domain‐general functional networks) we conducted a FNC analysis using the Mancovan toolbox in GIFT. Hence, FNC was estimated as the Pearson's correlation coefficient between pairs of time courses (Jafri et al., 2008). Subject‐specific time courses were detrended and despiked based on the median absolute deviation as implemented in 3dDespike (http://afni.nimh.nih.gov/), then filtered using a fifth‐order Butterworth low‐pass filter with a high‐frequency cutoff of 0.15 Hz. Pairwise correlations were computed between time courses, resulting in a symmetric *c*1 × *c*1 correlation matrix for each subject. For all FNC analyses, correlations were transformed to *z*‐scores using Fisher's transformation, *z* = atanh(*k*), where *k* is the correlation between two component time courses. One sample *t‐*test (corrected for multiple comparisons at α = .01 significance level, using false discovery rate) were conducted on task‐related functional networks to reveal the significance of pairwise correlations.

## 3 RESULTS

### 3.1 Behavioural results

Percentage of responses given revealed a non‐significant effect of “Task” [*F* (1, 44) = 0.163, *p* = .688, *ηp* ^2^ = .004], indicating that participants gave the same number of responses in the semantic (mean = 79.3%, SD = 13) and non‐semantic tasks (mean = 77.6%, SD = 13.9).

**FIGURE 2:**
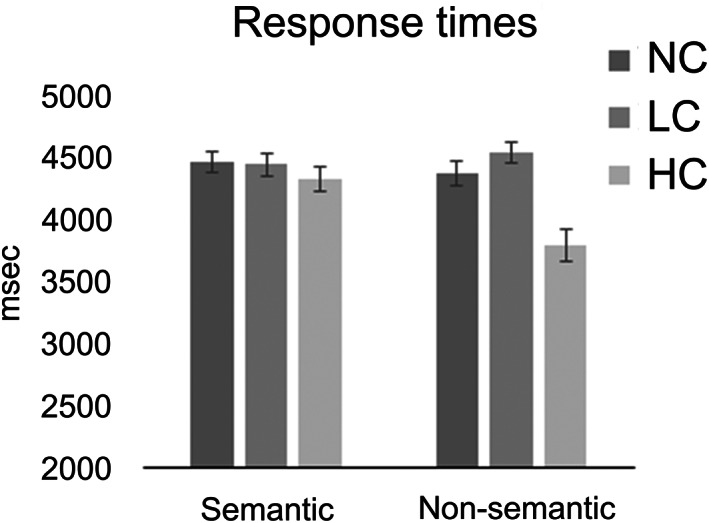
Behavioural results. RTs in semantic and non‐semantic tasks for NC (no‐context), LC (low congruent) and HC (high congruent) target conditions. Error bars correspond to Standard Errors.

The RT data revealed a non‐significant main effect of “Task” [*F* (1, 44) = 1.930, *p* = .172, *ηp* ^2^ = .042] but a significant effect of “Condition” [*F* (1.834, 80.708) = 40.555, *p* < .001, *ηp* ^2^ = .480]: NC and LC conditions were both slower than HC conditions (*p* = .008 and *p* = .019, respectively), whilst NC and LC conditions did not differ (*p* > .999). A significant interaction between “Condition” and “Task” [*F* (1.834, 80.708) = 20.575, *p* < .001, *ηp* ^2^ = .319] revealed some RT differences between conditions in the two tasks (see Figure 2).

Importantly and in line with the intended experimental design, a significant main effect of “Condition” was observed in both tasks. An ANOVA applied to data from the semantic task, revealed that conditions differed [*F* (1.982, 41.614) = 5.491, *p* = .008, *ηp* ^2^ = .207]: the HC condition was faster than both LC and NC conditions (*p* = .05 and *p* = .02, respectively), whilst LC and NC did not differ (*p* > .999). A second ANOVA, including data only from the non‐semantic task, also revealed a main effect of “Condition” [*F* (1.79, 41.179) = 38.561, *p* < .001, *ηp* ^2^ = .626]: NC and LC conditions elicited slower responses than HC conditions (both *ps* < .001), whilst LC and NC did not differ (*p* = .101).

### 3.2 fMRI results

#### 3.2.1 GLM results

##### Whole brain results

###### Context integration in semantic and non‐semantic tasks

We first identified which brain regions were engaged during contextual integration, irrespective of task (main effect and conjunction). Then, we examined which brain regions showed an interaction with the task.

**FIGURE 3:**
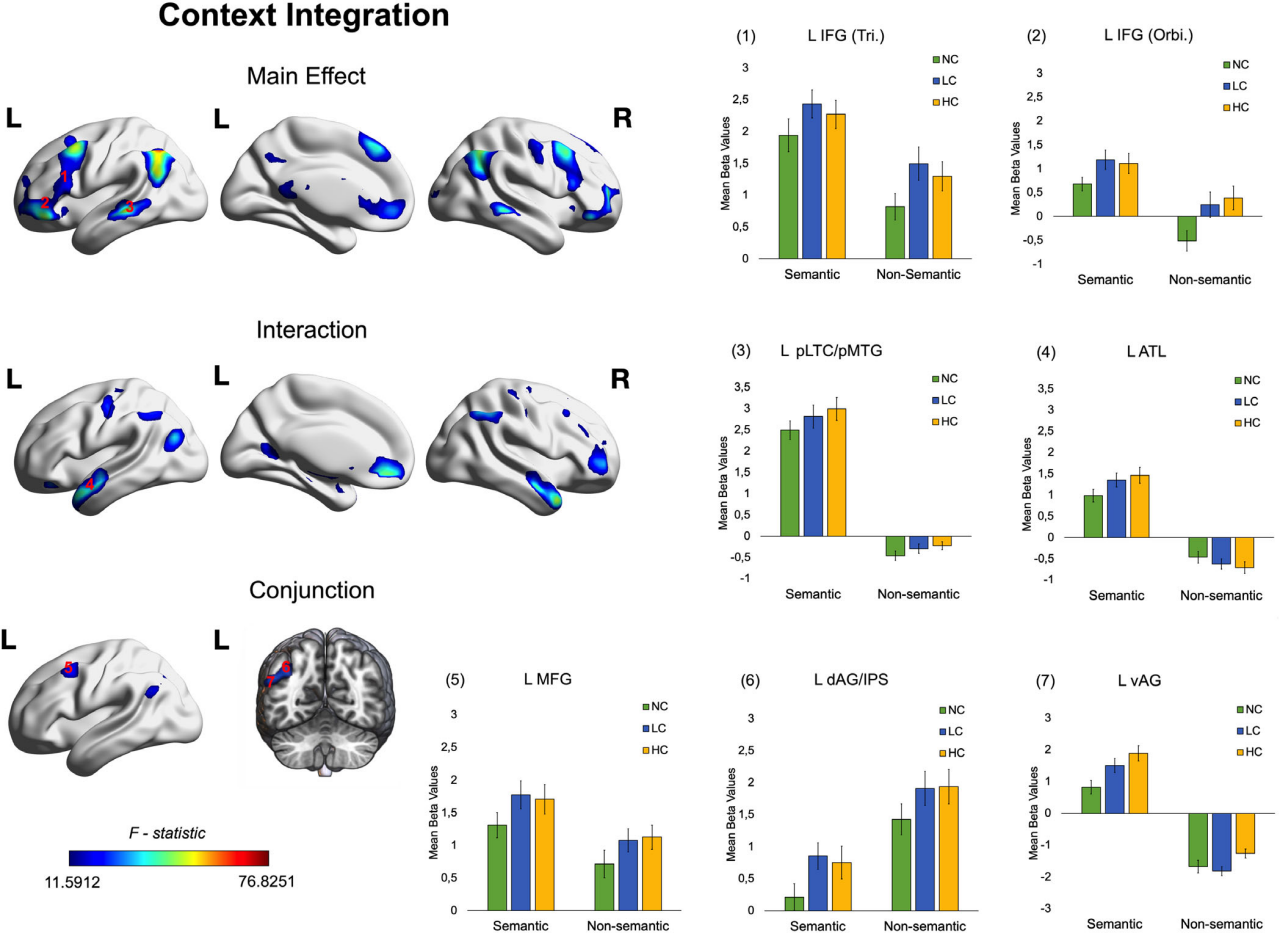
Main effect, interaction, and conjunction analysis results for the Context Integration effect (HC and LC vs. NC) in semantic and non‐semantic tasks. *F‐*maps were corrected for multiple comparisons using a voxel‐wise level significance threshold set at *p* < .001 with an FWE correction applied at the critical cluster level at *p* < .05. ROI results reflect mean beta values extracted from frontal, parietal and temporal brain regions. The precise MNI coordinates are derived from Jackson (2021) (see Table 1). Error bars correspond to Standard Errors. Abbreviations: ATL, anterior temporal lobe; dAG/IPS, dorsal angular gyrus/intra‐parietal sulcus; HC, high congruent; IFG, inferior frontal gyrus; L, left; LC, low congruent; MFG, middle frontal gyrus; NC, no‐context; Orbi., Pars Orbitalis; pLTC/pMTG, posterior lateral temporal cortex/posterior middle temporal gyrus; R, right; Tri., Pars Triangularis; vAG, ventral angular gyrus.

The main effect of "Context Integration" (HC and LC vs. NC) revealed significant neural activity in the expected frontoparietal network (see Figure 3 and Table S5 **)**, as well as in other brain regions, such as the ventromedial prefrontal cortex, a core area of the default mode network. Formal conjunction analysis for the same contrast revealed a cluster including both ventral and dorsal portions of the left AG (peak of maximal activity corresponding to the MNI coordinates: *x* = −39, *y* = −60, *z* = 36) which was similarly involved in semantic and non‐semantic context integration (see Figure 3).

###### Context update in semantic and non‐semantic tasks

Then, we explored which brain regions are important when it is hard to generate contextually integrated representations (LC) in general (i.e., irrespective of task) (main effect) and which brain regions, instead, show task conditional effects (interaction).

**FIGURE 4:**
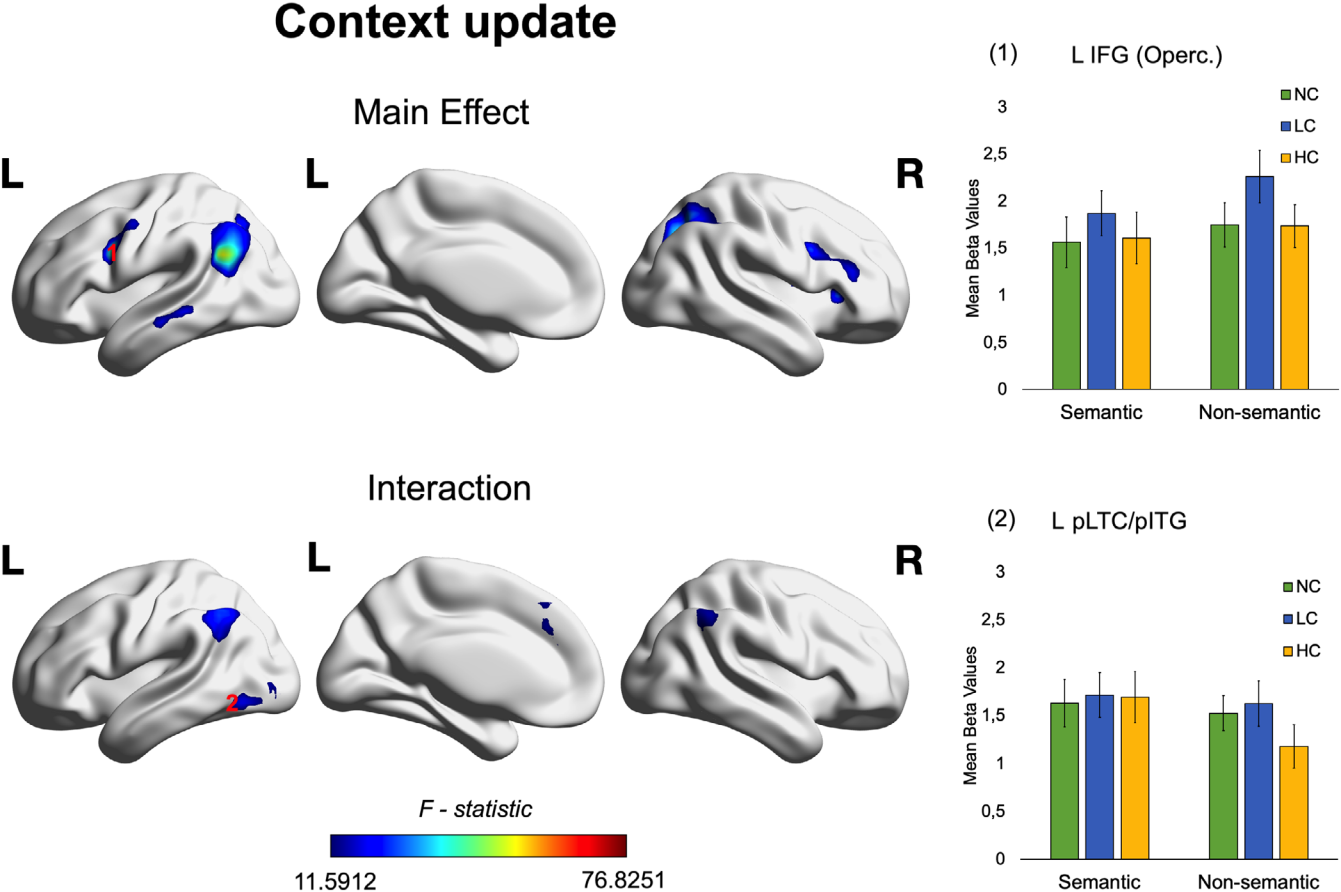
Main effect and interaction for the Context Update effect (LC vs. HC) in semantic and non‐semantic tasks. *F‐*maps were corrected for multiple comparisons using a voxel‐wise level significance threshold set at *p* < .001 with an FWE correction applied at the critical cluster level at *p* < .05. ROI results reflect mean beta values from left inferior frontal gyrus ‐ Pars Opercularis (IFG Operc.) and the left posterior lateral temporal cortex/posterior inferior temporal gyrus (L pLTC/pITG). The precise MNI coordinates are derived from Jackson (2021) (see Table 1). Error bars correspond to Standard Errors. Abbreviations: HC, high congruent; L, left; LC, low congruent; NC, no‐context; R, right.

The main effect of "Context Update" or *hard* contextual integration (LC vs. HC) revealed significant neural activity in superior parietal cortex, left ventral AG, and dorsal AG/IPS (see Figure 4 and Table S5). The results also revealed significant neural responses in precentral gyrus, right insula (anterior and posterior), as well as right putamen (see Figure 4 and Table S5). These regions, however, were not recruited to the very same extent in the two tasks, as indicated by non‐significant conjunction analysis results.

Interestingly, the interaction results revealed significant neural activity in bilateral inferior parietal lobes, frontal superior medial lobe, and left inferior posterior occipitotemporal cortex (see Figure 4 and Table S5).

##### ROI results

The goal of this analysis was to unpack and visualise the task‐engagement profile of key frontal, parietal, and temporal brain regions revealed in the whole‐brain results. Discrete regions within left IFG, left pLTC and left AG, might show functional dissociations depending on the type of stimuli and task difficulty (see “Introduction”). Accordingly, we selected different frontal (left IFG Pars Opercularis, Pars Triangularis, and Pars Orbitalis), temporal (left pLTC/pMTG and left pLTC/pITG) and parietal (anterior ventral AG and dorsal AG/IPS) ROIs for these analyses (see Table 1), and then we compared the patterns of activation across semantic and non‐semantic tasks.

**TABLE 1:** Results from the independent region of interest (ROI) analysis.

ROIs	Specific coordinate source in Jackson (2021)	MNI coordinate			ANOVA results			
		*x*	*y*	*z*	Area	Condition	Task	Interaction
L IFG (Pars Triangularis; see Figure 3)	*Semantic control activation likelihood*. Peaks coordinates are reported in Table 1 and at https://github.com/JacksonBecky/SemanticControlMetaA)	−48	22	20	Area: *F* (1.731, 76.184) = 28.145, *p* < .001, η*p*2 = .39	Condition: *F* (1.648, 72.515) = 18.943, *p* < .001, η*p*2 = .301	Task: *F* (1, 44) = 6.064, *p* = .018, η*p*2 = .121	Area × Task: *F* (1.731, 76.184) = 6.775, *p* = .003, η*p*2 = .133
L IFG (Pars Orbitalis; see Figure 3)		−46	40	−10	L IFG Tri. > L IFG Orbi. (*p* < .001) L IFG Operc. > L IFG Orbi. (*p* < .001) L IFG Tri. versus L IFG Operc. (*p* > .999)	LC versus HC (*p* = .137) HC and LC > NC (both *ps* < .001)	Semantic > Non‐semantic	L IFG Tri.: Semantic > Non‐semantic (*p* = .032) L IFG Orbi.: Semantic > Non‐semantic (*p* = .057) Lef IFG Operc.: Semantic versus non‐semantic (*p* > .999)
L IFG (Pars Opercularis; Figure 4)		−44	8	24				Condition × Task: *F* (1.648, 72.515) = .807, *p* = .429, η*p*2 = .018
								Area × Condition: *F* (3.130, 137.728) = 13.781, *p* < .001, η*p*2 = .239.
								L IFG Tri.: LC versus HC (*p* > .999); HC and LC > NC (*p* = .006 and *p* < .001); Left IFG Orbi.: LC versus HC (*p* > .999); HC and LC > NC (both *ps* < .001) L IFG Operc.: LC versus HC (*p* = .007); HC and LC > NC (*p* > .999 and *p* = .004)
								Area × Condition × Task: *F* (3.130, 137.728) = 2.715, *p* = .045, η*p*2 = .058.
L anterior vAG (see Figure 3)	*Semantic cognition activation likelihood*. Peaks coordinates are reported in Tables 3, S3, and at https://github.com/JacksonBecky/SemanticControlMetaA	−46	−66	26	Area: *F* (1, 44) = 48.239, *p* < .001, η*p*2 = .523	Condition: *F* (1.766, 77.709) = 28.121, *p* < .001, η*p*2 = .39	Task: *F* (1, 44) = 15.573, *p* < .001, η*p*2 = .261	Area × Task: *F* (1, 44) = 127.439, *p* < .001, η*p*2 = .743
L dAG/IPS (see Figure 3)		−32	−66	40	L dAG/IPS > L anterior vAG	LC < HC (*p* = .039); HC and LC > NC (both *ps* < .001)	Semantic > Non‐semantic	L anterior vAG: Semantic > Non‐semantic (*p* < .001) L dAG/IPS: Non‐semantic > Semantic (*p* = .001)
								Condition × Task: *F* (1.766, 77.709) = 4.284, *p* = .021, η*p*2 = .089
								Semantic: HC and LC > NC (both *ps* < .001); HC versus LC (*p* > .999) Non‐semantic: LC versus NC (*p* > .999); HC > NC (*p* = .003); LC versus HC (*p* = .23)
								Condition × Area: *F* (1.899, 83.575) = 12.229, *p* < .001, η*p*2 = .217
								L anterior vAG: LC versus NC (*p* = .119); HC > NC (*p <* .001); HC > LC (*p <* .001) L dAG/IPS: LC > NC (*p* < .001); HC > NC (*p* < .001); LC versus HC (*p* > .999)
								Condition × Area × Task: *F* (1.899, 83.575) = 6.481, *p* = .003, η*p*2 = .128
								L anterior vAG and Semantic: LC versus HC (*p* = .587); LC > NC (*p* < .001); HC > NC (*p* < .001); L anterior vAG and Non‐semantic: LC < HC (*p* = .006); LC versus NC (*p* > .999); HC versus NC (*p* = .205); L dAG/IPS and Semantic: LC versus HC (*p* > .999); LC > NC (*p* < .001); HC > NC (*p* = .017); L dAG/IPS and Non‐semantic: LC versus HC (*p* > .999); LC > NC (*p* = .042); HC > NC (*p* = .020)
L pLTC/pMTG (Figure 3)	*Semantic control activation likelihood*. Peaks coordinates are reported in Table 1 and at https://github.com/JacksonBecky/SemanticControlMetaA	−54	−42	4	Area: *F* (1, 44) = 2.879, *p* = .097, η*p*2 = .061	Condition: *F* (1.811, 79.678) = 2.290, *p* = .113, η*p*2 = .049	Task: *F* (1, 44) = 65.422, *p* < .001, η*p*2 = .598	Area × Task: *F* (1, 44) = 49.659, *p* < .001, η*p*2 = .530
L pLTC/pITG (see Figure 4)		−46	−56	−12			Semantic > Non‐semantic	L pLTC/pITG Non‐semantic versus Semantic (*p* > .999); L pLTC/pMTG: Semantic > Non‐semantic (*p* < .001);
								Condition × Task: *F* (1.811, 79.678) = 2.499, *p* = .094, η*p*2 = .054
								Condition × Area: *F* (1.975, 86.897) = 21.66, *p* < .001, η*p*2 = .33
								L pLTC/pITG: NC versus LC (*p* > .999); NC versus HC (*p* > .999); LC versus HC (*p* = .131); L pLTC/pMTG: NC versus LC (*p* = .113); NC < HC (*p* < .001); LC versus HC (*p* > .999)
								Condition × Area × Task: *F* (1.975, 86.897) = 1.981, *p* = .145, η*p*2 = .043
Left ATL (see Figure 3)	*Semantic cognition activation likelihood*. Peak coordinate is reported in Table 3 and at https://github.com/JacksonBecky/SemanticControlMetaA	−52	8	−18	N.A.	Condition: *F* (1.805, 79.4) = 0.794, *p* = 0.44, η*p*2 = .018	Task: *F* (1, 44) = 106.077, *p* < .001, η*p*2 = 0.707	Condition × Task: *F* (1.805, 79.4) = 6.796, *p* = .003, η*p*2 = 0.134
							Semantic > Non‐semantic	Semantic: HC and LC versus NC (*p* = .023 and *p* = .221); LC versus HC (*p* > .999) Non‐semantic: LC versus NC (*p* > .999); HC versus NC (*p* > .999); HC versus LC (*p* > .999)
Left MFG (see Figure 3)	*Semantic cognition activation likelihood*. Peak coordinate is reported in Table 3 and at https://github.com/JacksonBecky/SemanticControlMetaA	−39	6	48	N.A.	Condition: *F* (1.694, 74.532) = 16.163, *p* < .001, η*p*2 = 0.269	Task: *F* (1, 44) = 5.106, *p* = .029, η*p*2 = .104	Condition × Task: *F* (1.694, 74.532) = 0.297, *p* = .707, η*p*2 = .007
						LC versus HC (*p* > 0.999); HC and LC > NC (both *ps* < .001)	Semantic > Non‐semantic	

*Note*: The MNI coordinates for each anatomical area of interest correspond to peaks of activation derived from Jackson (2021). Bonferroni correction for multiple comparisons was applied to all post‐hoc comparisons. Bonferroni‐corrected *p*‐values are reported accordingly. Furthermore, correction for non‐sphericity (Greenhouse–Geisser procedure) was applied to the degrees of freedom and *p*‐values associated with factors having more than two levels.

Abbreviations: ATL, anterior temporal lobe; dAG/IPS, dorsal angular gyrus/intra‐parietal sulcus; HC, high congruent; IFG, inferior frontal gyrus; L, left; LC, low congruent; MFG, middle frontal gyrus; NC, no‐context; Operc., Pars Opercularis; Orbi., Pars Orbitalis; pITG, posterior inferior temporal gyrus; pLTC, posterior lateral temporal cortex; pMTG, posterior middle temporal gyrus; R, right; Tri., Pars Triangularis; vAG, ventral angular gyrus.

Finally, we also computed ROI analysis on middle frontal gyrus (MFG) and ATL revealed by whole‐brain results (see Table 1). Note that in this case our goal was not to compare these brain regions to each other, but just to examine the pattern of activations within each ROI for semantic versus non‐semantic tasks. Thus, we ran two separate ANOVAs.

The ROI results are reported in Table 1, and Figures 3, 4. To summarise the most important results, we found that different left IFG subregions were engaged to different extents in semantic and non‐semantic tasks. That is, left IFG Pars Triangularis and Pars Opercularis were both positively recruited in both tasks, for context integration and context update, respectively. Instead, left IFG Pars Orbitalis showed domain‐specific responses: A positive pattern of engagement was found in the semantic task, but not in the non‐semantic task. In this latter, left IFG Pars Orbitalis's neural responses were only negative (NC) or negligible (LC and HC), as revealed by one sample *t‐*tests (NC: *t* (23) = −2.304, *p* = .031; LC: *t* (23) = 0.897, *p* = .379; HC: *t* (23) = 1.491, *p* = .149) (see Figure 3).

A similar dissociation was found in the left AG (see Figure 3). Left anterior ventral AG was more engaged for semantic versus non‐semantic task conditions. Instead, the dorsal AG/IPS showed the opposite pattern of responses. It is important to note that, however, whilst the left ventral AG was positively engaged in semantic task conditions only (and de‐activated in non‐semantic task conditions), the left dorsal AG/IPS was positively recruited in both tasks for context integration, therefore confirming the domain‐general role of this region for buffering contextual information. Finally, we found that MFG and ATL were both more engaged in semantic versus non‐semantic task conditions (see Table 1).

#### 3.2.2 Task group spatial ICA results

##### Task‐related functional networks

**FIGURE 5:**
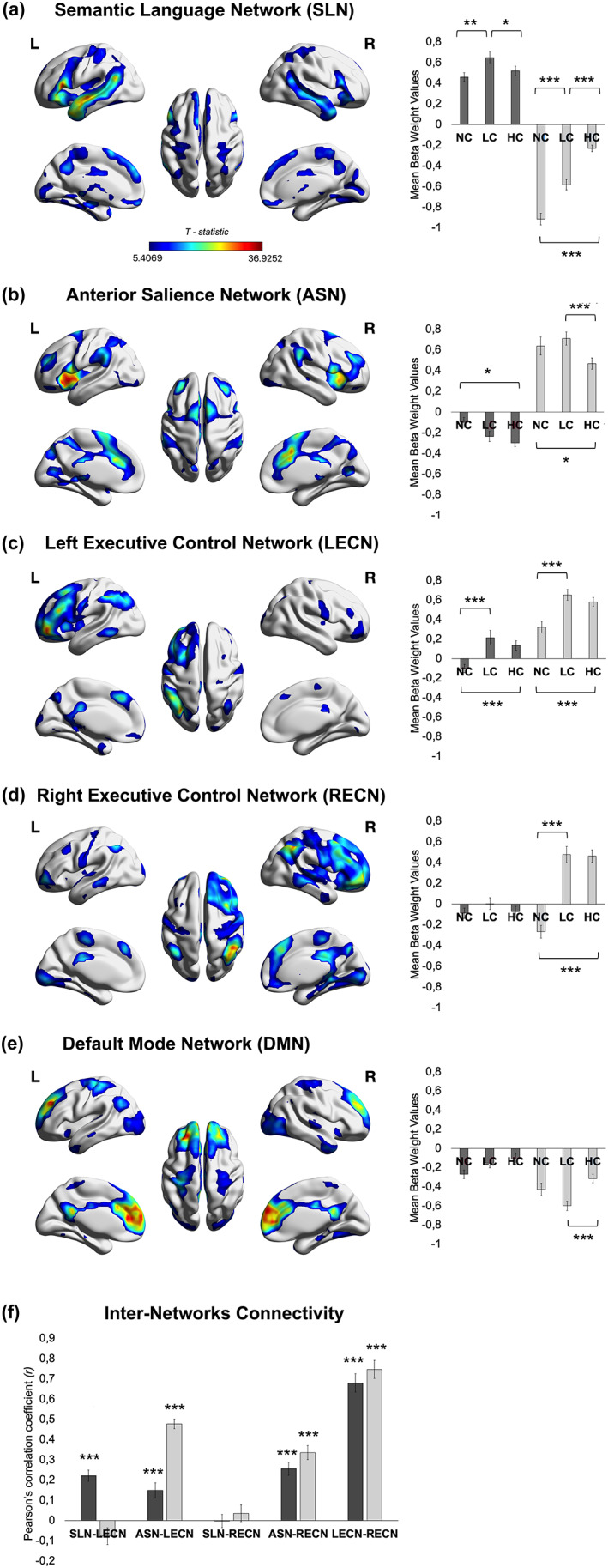
Task‐related functional networks. (a) Semantic/Language Network (SLN); (b) Anterior Salience Network (ASN); (c) left Executive Control Network (LECN); (d) right Executive Control Network (RECN); (e) Default‐Mode Network (DMN). ROI results reflect the mean beta weight values for each condition (NC, LC, and HC), where dark grey bars and light grey bars refer to semantic and non‐semantic tasks, respectively. Error bars correspond to Standard Errors. (f) Inter‐Networks Connectivity for each task. Asterisks positioned over the bars indicate significant inter‐network correlations. Dark grey bars and light grey bars refer to semantic and non‐semantic tasks, respectively. Error bars correspond to Standard Errors. Abbreviations: HC, high congruent; L, left; LC, low congruent; NC, no‐context; R, right.

ICA identified 26 independent components, of which five exhibited significant sensitivity to the task conditions in both semantic and non‐semantic tasks: these were a semantic/language network, an anterior salience network, left and right executive control networks, both including frontoparietal regions. Finally, ICA identified also a default mode network component (see Figure 5 and Table S6).

A first observation is that the spatial maps of left and right executive control networks include similar brain regions as those identified with the context integration univariate results. We conducted a repeated‐measures ANOVA on the beta weight values of each component to examine context integration and context update across tasks. The results revealed a significant interaction between “Network” (semantic/language network, left anterior salience network, left and right executive control networks, and default mode network), “Condition” (NC, LC, and HC), and “Task” (semantic and non‐semantic), which suggests a different engagement of the networks in the different conditions and tasks [*F* (4.624, 203.468) = 18.337, *p* < .001, *ηp* ^2^ = .294].

The semantic/language network was differently engaged in the two tasks (see Figure 5 and Table S6). In the semantic task, the semantic/language network showed increased responses for LC conditions as compared to both HC and NC conditions (*p* = .035 and *p* = .005, respectively), whilst the difference between HC and NC was not significant (*p* = .895). In the non‐semantic task, there were decreased, rather than increased, responses in semantic/language network as compared to the rest baseline. Thus, HC conditions showed less deactivation as compared to LC and NC conditions (both *ps* < .001), whilst the LC conditions showed less deactivation than the NC conditions (*p* < .001).

The anterior salience network showed an opposite pattern of results to the semantic/language network. Neural responses were positive in the non‐semantic task, but negative in the semantic task. In the semantic task, NC conditions differed from HC conditions (*p* = .013). There were no significant differences between LC and HC conditions (*p* = .436), nor between LC and NC conditions (*p* = .124). In the non‐semantic task, NC and LC conditions showed more positive responses as compared to HC conditions (*p* = .030 and *p* < .001), whilst the LC and NC conditions did not differ (*p* = .757).

The left executive control network was similarly engaged in the two tasks. In the semantic task, it showed increased positive responses when the semantic context was available for integration (LC > NC and HC > NC, both *ps* < .001), but no difference between LC and HC was observed (*p* = .471). Also in the non‐semantic task, increased positive responses were observed when context integration was possible (LC > NC and HC > NC, both *ps* < .001). As above, no difference between LC and HC was observed (*p* = .515). This domain‐general pattern of responses for context integration is in line with the conjunction analysis results. In fact, the left executive control network spatially overlaps with the brain regions that were similarly activated in semantic and non‐semantic tasks during context integration.

The right executive control network showed a similar context integration effect as for the left executive control network, but only in the non‐semantic task (HC > NC and LC > NC, both *ps* < .001; HC vs. LC, *p >* .999).

Finally, unlike the other components, the default mode network was deactivated with respect to the rest baseline. Conditions differed only in the non‐semantic task and reflected differential de‐activations (non‐semantic task: NC vs. LC, *p* = .054; NC vs. HC, *p* = .318; HC > LC, *p* < .001).

##### FNC analysis

Given our interest in exploring functional interactions between the domain‐specific (semantic/language network, anterior salience network, and right executive control network) and domain‐general networks (left executive control network), we computed an FNC analysis (see Figure 5f). An intriguing pattern of results emerged. The strength of correlations between domain‐specific and domain‐general networks changed as a function of the task, as revealed by repeated‐measures ANOVA's results. In detail, in this analysis “Task” was a factor with two levels (semantic and non‐semantic), and “Type of FNC” a factor with five levels (semantic/language network—left executive control network; semantic/language network—right executive control network; anterior salience network—left executive control network; anterior salience network—right executive control network; and left executive control network—right executive control network). Bonferroni correction for multiple comparisons was applied to assess significant effects. Bonferroni‐corrected *p*‐values were reported accordingly. Correction for non‐sphericity (Greenhouse–Geisser procedure) was applied to the degrees of freedom and *p‐*values associated with factors having more than two levels.

The results revealed a significant interaction between “Task” and “Type of FNC” [*F* (2.566, 112.886) = 19.822, *p* < .001, *ηp* ^2^ = .311], indicating that the significant positive correlation observed between time courses of left executive control network and semantic/language network was stronger in the semantic task versus the non‐semantic task (*p* < .001). Instead, the opposite pattern was observed for left executive control network—anterior salience network coupling. That is, stronger positive correlation in the non‐semantic task as compared to the semantic task (*p* < .001). Interestingly, semantic/language network—right executive control network, anterior salience network—right executive control network, and left executive control network—right executive control network did not show any difference between tasks (*ps* > .999; see Figure 5f **)**.

#### 3.2.3 Summary of the results

The main effect of context integration revealed strong brain activations within the semantic control network (Jackson, 2021; Lambon Ralph et al., 2017), including left IFG, left pLTC, as well as left AG. A closer look at these results revealed dissociations in the patterns of engagement within these regions. For instance, left IFG (Pars Orbitalis), left anterior ventral AG, and left pLTC/pMTG were uniquely engaged during the integration of semantic information. Instead, left IFG Pars Triangularis and left dorsal AG/IPS did not show these domain‐specific effects, but rather a domain‐general role for generating context‐integrated representations.

Interestingly, the main effect of context update/hard contextual integration revealed significant activations in different portions of the frontal and parietal lobes, as compared to those revealed by the main effect of context integration. In fact, left posterior IFG (Pars Opercularis) as well as the superior parietal cortex were involved in updating the contextual representation, irrespective of the type of stimuli (Figure 4 and Table S5). Interestingly, the pLTC/pITG showed a context update effect but only for non‐semantic stimuli. Finally, the GLM and ROI results also revealed that left superior lateral ATL and left pMTG were uniquely involved in semantic contextual integration (see Figure 3 and Table 1).

ICA results showed that whilst the left executive control network supports domain‐general context integration, the update of contextual information is supported by domain‐specific networks (anterior salience network and semantic/language network). Interestingly, also a right executive control network is involved in context integration, but only for the generation of non‐semantic contextual representations. Finally, interactions between the left executive control network and domain‐specific networks are modulated by the type of task, and therefore, the nature of the stimuli involved during context integration.

## 4 DISCUSSION

The present fMRI study investigated the extent to which neural processes reflecting context integration and context update are domain‐specific versus domain‐general. Further exploration of the whole‐brain results via ROI analysis focused particularly on the role of different subregions of left IFG, left pLTC, and left AG. In fact, these regions are functionally heterogenous, with ongoing debates regarding (1) which sub‐regions are implicated in semantic processing and beyond (Diachek et al., 2020; Hodgson et al., 2021; Humphreys et al., 2021; Seghier, 2013; Seghier et al., 2010; Wehbe et al., 2021) and (2) the extent to which their involvement in semantic tasks reflects methodological artefacts (Fedorenko & Shain, 2021).

We hypothesised to find both shared (left IFG Pars Opercularis and Triangularis, left pLTC/pITG, and left dorsal AG/IPS) and distinct (left IFG Pars Orbitalis, pLTC/pMTG and anterior ventral AG for semantic stimuli only) brain regions involved in context integration and update. We found that left IFG, left pLTC, and left AG functionally fractionate, as they revealed both domain‐specific and domain‐general subregions for context integration and update. The results, which are discussed below, provide the first characterisation of the neural processes during naturalistic semantic processing along the domain‐specificity dimension, and at the same time, bring new insights into the role of left pLTC/pITG and left dorsal AG/IPS for semantic cognition.

### 4.1 Left IFG

The left IFG Pars Opercularis was sensitive to control demands during context integration (LC > HC), and this effect was independent of the type of task/stimulus. This is consistent with previous studies that have found left IFG Pars Opercularis to be more active when sentence meaning was ambiguous or implausible as compared to plausible (Desai et al., 2010; Obleser & Kotz, 2010; Rodd et al., 2005; Willems et al., 2009). This neural effect has been interpreted in many different ways (Price, 2012). Some have proposed that this could reflect processing violations of the “what” related predictions and integration/update of the input (Obleser & Kotz, 2010). Others have related this effect to increased verbal working memory demands (Koelsch et al., 2009). Our results do not seem to favour the latter hypothesis. In fact, we did not observe any difference between conditions that require (HC) versus those that do not require (NC) to maintain information in working memory to allow context integration. Thus, the context update effect observed in left IFG Pars Opercularis may reflect the consequences of the detection of prediction violation. Our data also show that left IFG Pars Opercularis was similarly engaged in semantic and non‐semantic tasks. This is in accord with studies that revealed incongruency effects in this sub‐region, not only in language comprehension but also in music and action perception (Bianco et al., 2016; Koelsch et al., 2002; Siman‐Tov et al., 2019; Tillmann et al., 2006).

Neural responses observed in left Pars Opercularis were quite specific for the LC condition (i.e., did not track task difficulty) and, at the same time, not restricted to the verbal/semantic domain. Therefore, it is likely that this subregion supports domain‐general processes for context integration, that might come into play only under increased task demands, for example, during the update or reset of an evolving representation.

Also left IFG Pars Triangularis was positively recruited by semantic and non‐semantic tasks, a result that aligns with our hypothesis that this brain region would reflect domain‐general processes for context integration. Indeed, previous studies have found that this subregion is not only implicated in the control of semantic information (Badre & Wagner, 2002; Gold et al., 2005, 2006). For instance, competition in episodic and working memory tasks also increases activation of left IFG Pars Triangularis (Badre & Wagner, 2002; Dobbins & Wagner, 2005).

Somewhat unexpectedly, the left IFG Pars Triangularis did not show enhanced neural responses during context update or LC conditions. Incongruency effects in left IFG Pars Triangularis have been mainly observed in tasks where retrieval of information occurred with little or no contextual support (Hodgson et al., 2021; Humphreys et al., 2020; Price, 2012; Rodd et al., 2005; Whitney, Jefferies, & Kircher, 2011; Whitney, Kirk, et al., 2011). Therefore, it is possible that those incongruency effects reflected task‐specific demands rather than semantic control processes typically required in context integration itself.

As expected, left IFG Pars Orbitalis exhibited domain (semantic)‐specific responses and was sensitive to context integration (HC and LC > NC). This observation is in accordance with a recent meta‐analysis study where left IFG Pars Orbitalis—unlike Pars Opercularis and Triangularis—was modulated by semantic control demands but not by other types of non‐semantic demands (Hodgson et al., 2021). Accordingly, this anterior and ventral subregion of the left IFG lies outside the multiple demand network (Assem et al., 2020; Duncan, 2010; Duncan et al., 2020; Fedorenko et al., 2013). Thus, left IFG Pars Orbitalis may be specialised for the control of semantic information (Badre et al., 2005; Badre & Wagner, 2007; Devlin et al., 2003; Gough et al., 2005; Wagner et al., 2001). In contrast, more posterior‐dorsal subregions (Pars Triangularis and Pars Opercularis) may support domain‐general computations. Alternatively, left IFG's activity would reflect the same neuro‐computation (e.g., context integration) and the graded differences might depend on variations in functional and structural connectivity with areas and/or networks that support domain‐specific processes. For instance, left IFG Pars Orbitalis is directly connected via uncinate fasciculus to the ATL (Binney et al., 2012), a neural hub for semantic representations (Lambon Ralph et al., 2017; Patterson et al., 2007). Instead, left IFG Pars Opercularis and Triangularis are directly connected to brain regions of the multiple demand network, such as the dorsolateral prefrontal cortex (Jung et al., 2022), which are activated across different domains when task demands increase. In our data, left IFG activations during context integration and update overlapped substantially across tasks. This was found not only in the whole brain results (ANOVA), but also in the functional networks revealed by ICA, where domain‐specific (semantic/language network and anterior salience network) and domain‐general networks (left executive control network) overlapped in left IFG, a result that does not align neatly with a semantic versus domain‐general distinction for the functional role of left IFG.

### 4.2 Left pLTC

Whole‐brain analyses revealed that left pLTC/pMTG responded similarly to context integration in semantic and non‐semantic tasks, yet it was positively engaged in the semantic task only. Accordingly, left pLTC/pMTG was embedded in the semantic‐specific network (semantic/language network), but not in the network showing domain‐general effects (e.g., left executive control network). These results are in accordance with our hypothesis that left pLTC/pMTG would be specialised for the integration of semantic information (Hodgson et al., 2021; Humphreys & Lambon Ralph, 2017). Like left IFG Pars Orbitalis, left pLTC/pMTG did not show increased neural responses when integration demands increased (LC condition). It is possible that this effect was not observed because of differences between our and previous studies (e.g., semantic judgement task, thematic association task; Whitney, Kirk, et al., 2011). In our semantic task, participants were provided with rich semantic contexts, which is very different from previous paradigms where little contextual information was provided during semantic retrieval, potentially increasing the control demands by necessitating additional context‐relevant information generation and retrieval.

Interestingly, whole‐brain results revealed a completely different pattern of engagement in left pLTC/pITG. This subregion was engaged to the same extent in semantic and non‐semantic tasks. However, our results revealed a role for this subregion in context updates during the processing of numerical, but not semantic stimuli. This finding, which is at odds with our initial hypothesis, that is, to find this subregion similarly engaged in semantic and non‐semantic tasks for context update (Hodgson et al., 2021), suggests that left pLTC/pITG may not be recruited for semantic control, but only as part of the multiple demand network when the task loads strongly on executive control (Fedorenko & Shain, 2021). This proposal is also consistent with the fact that left pLTC/pITG was functionally embedded in the left executive control network but not the semantic/language network.

### 4.3 Left AG

Whole‐brain results revealed a functional overlap across tasks in the left AG, and especially left dorsal AG/IPS, implicating this region in context integration processes beyond the semantic domain. Accordingly, left dorsal AG/IPS was recruited within a frontoparietal network (left executive control network), which was sensitive to context integration in both tasks.

We also revealed some domain‐specific responses within the left AG. As expected, left anterior ventral AG was positively engaged (as compared to rest) only when context integration involved semantic stimuli. Note that the dissociation observed between neural responses in left dorsal AG/IPS and left anterior ventral AG cannot be related to differences in task difficulty, as there was no RT difference between the two tasks. Furthermore, when positively activated by the task, both subregions showed context integration effects that did not track difficulty‐related differences between conditions. For instance, a difference between LC and NC conditions is observed in both left AG subregions. However, the same effect is absent in RTs. This result, which is not necessarily in contrast with previous evidence showing difficulty effects in the AG (Humphreys & Lambon Ralph, 2017; Kuhnke et al., 2023; Zhang et al., 2023), suggests that the context effects observed in the left AG cannot be easily explained as due to differences in task difficulty between conditions.

The domain‐specific and domain‐general (conjunction results) neural results presented above are consistent with the proposal that the core function of left AG is a functionally‐graded domain‐general buffer, with subregions showing different patterns of engagement depending on their pattern of connectivity to various domain‐specific systems (Humphreys et al., 2022; Humphreys & Lambon Ralph, 2015). Accordingly, left anterior ventral AG, which is functionally and anatomically connected to the language network (see semantic/language network; Humphreys et al., 2022; Makris et al., 2013), showed strong positive responses during the integration of verbal, but not numerical information.

We also tested the hypothesis of differential involvement of dorsal AG/IPS and anterior ventral AG regions depending on the context integration demands. Specifically, we hypothesised that an update of context (LC > HC) would require top‐down control processes, implemented by left dorsal AG/IPS (Corbetta & Shulman, 2002; Humphreys & Lambon Ralph, 2015, 2017; Noonan et al., 2013). Conversely, we predicted stronger neural responses for HC versus LC conditions in left anterior ventral AG, reflecting automatic bottom‐up buffering processes (Humphreys & Lambon Ralph, 2015). Interestingly, we observed stronger responses for HC as compared to LC conditions in left anterior ventral AG, but we did not observe stronger responses in left dorsal AG/IPS for the opposite contrast (see Figure 3). One possibility is that left dorsal AG/IPS's sensitivity to task demands reported by previous studies did not primarily reflect buffering‐related neural processes, but rather top‐down attentional control processes imposed by a secondary task (e.g., a memory probe, comprehension, or sentence judgement task).

Overall, the findings reviewed above are in accordance with previous reports showing a link between left AG's activity and the integration of contextual information in language tasks (Branzi, Humphreys, et al., 2020; Branzi, Pobric, et al., 2021; Humphreys et al., 2020; van der Linden et al., 2017). The current study goes further, by establishing that left dorsal AG/IPS reflects domain‐general processes for context integration (Humphreys et al., 2020; Humphreys & Lambon Ralph, 2015, 2017; Ramanan & Bellana, 2019).

Our results have implications for the debate on the role of left AG in semantic cognition. In fact, in contrast with the proposal of the domain‐general buffer's role, other researchers have argued that left AG would reflect a supramodal conceptual semantic hub (Binder et al., 2009). Our results are not compatible with this view. In fact, conjunction analysis results revealed that left AG, and especially dorsal AG/IPS was recruited to the same extent in both tasks.

### 4.4 Domain‐general and domain‐specific functional networks

The ICA results revealed a frontoparietal network (left executive control network), including working memory as well as multiple demand network brain areas (Hodgson et al., 2021). The left executive control network was similarly recruited for semantic and non‐semantic context integration. In contrast, a language network (i.e., semantic/language network) including anterior and posterior temporal areas, left anterior ventral AG, and left IFG, was positively engaged during the semantic task, especially for context update. This network was suppressed during the non‐semantic task. ICA revealed another domain‐specific network, the anterior salience network, primarily composed of the anterior insula and dorsal anterior cingulate cortex (Menon, 2011; Menon & Uddin, 2010). This network was strongly engaged during the non‐semantic task for context update but suppressed during the semantic task. Finally, our results revealed that interactions between the left executive control network and the domain‐specific networks (i.e., semantic/language network and anterior salience network) were modulated by the type of task, and therefore, the nature of the stimuli involved in context integration.

The results from the ICA complement the whole‐brain results and deepen our understanding of the neural mechanisms behind context integration and update. The left executive control network, consisting of frontoparietal brain regions, overlaps with left AG and MFG revealed by the conjunction analysis results (see Figure S4), that is, brain regions commonly activated by both tasks during context integration. Thus, the involvement of this network in both tasks likely reflects domain‐general processes that assist in the construction of contextual representations. We propose that this network is engaged during online buffering and integration of information over time. This left executive control network also interacts with other two domain‐specific networks, the anterior salience network and the semantic/language network, depending on the task. It has been suggested that the anterior salience network plays an important role in the detection of unexpected or salient stimuli and the subsequent engagement of the left executive control network for working memory and higher‐order cognitive control (Menon, 2011). In contrast, the semantic/language network has been associated with semantic‐specific neurocomputations, including tracking of coherence and the detection of semantic violations (Branzi, Humphreys, et al., 2020; Diachek et al., 2020; Fedorenko & Shain, 2021). The semantic/language network's activity is also accompanied by the activity of frontoparietal brain regions, especially when task demands increase. Accordingly, it is possible that the anterior salience network and semantic/language network detect violations of domain‐specific expectations. Then, the need to update the ongoing representation might determine the additional recruitment of the left executive control network, which would aid the integration and update of the ongoing representations within domain‐specific areas (e.g., ATL). The observed interactions between domain‐specific networks and left executive control network are in accordance with the hypothesis that domain‐specific and domain‐general networks work in tandem for context integration.

Finally, ICA also revealed a right executive control network, which is bilateral yet asymmetric, unlike the left executive control network which is more strongly left lateralized. This network was uniquely engaged in the non‐semantic task, in accord with the lack of functional interactions between the right executive control network and the semantic/language network. It is possible that the non‐semantic task requires processes not shared with the linguistic domain for the integration of numerical information. For instance, unlike the semantic task, the numerical task requires arithmetic calculations. This might explain the hemispheric fractionation (right executive control network and left executive control network) of the executive control network in the non‐semantic task only.

### 4.5 Limitation

Although it is unlikely that gender differences have affected the neural results (e.g., Eliot et al., 2021), gender disparity in our sample might represent a limitation for generalising the present results to non‐female populations.

### 4.6 Conclusion

Our results suggest that semantic control and domain‐general control are partially dissociable (IFG, AG, and pLTC), and thus that semantic control processes are not fully overlapping with other types of cognitive demands (e.g., Gao et al., 2021). Using ICA, we revealed that despite the left executive control network and semantic/language network being both engaged and interacting during semantic processing, only the latter reflects core‐linguistic operations (e.g., update of the semantic gestalt). Thus, the left executive control network may work together with the semantic/language network, carrying out general operations (the same as in the non‐semantic tasks) on semantic‐specific knowledge representations. In addition, our results indicate that these control systems are highly interactive. This is in line with multiple lines of research. For instance, after a stroke, domain‐general executive function skills are important predictors of the aphasia recovery (Geranmayeh et al., 2017). Furthermore, stimulating regions from the multiple demand network improves language acquisition (Sliwinska et al., 2017), a finding which is consistent with the evidence of transfer effects between language control and domain‐general executive control in healthy and patient populations (e.g., Cattaneo et al., 2015; Timmer et al., 2019). Our findings complement this evidence by showing that during naturalistic language processing, the semantic/language network and the left executive control network strongly interact. Future studies are needed to clarify the functional relevance of these interactions, and *if* and *when* these interactions become *necessary* for language and semantic cognition.

## CONFLICT OF INTEREST STATEMENT

The authors declare no conflicts of interest.

## Supporting information


**Table S1.** Stimuli for the semantic task. Stimuli for low congruent (LC) and high congruent (HC) conditions (context and target paragraphs). The shift of semantic context after low‐congruent contexts was expected to be perceived after the critical ambiguous word, here depicted in red.
**Table S2.** Stimuli for the comprehension task (semantic task).
**Table S3.** Stimuli for the non‐semantic task. The labels for LC and HC conditions (context and target paragraphs) refer to .bmp files that can be downloaded from the “Non‐semantic_task_stimuli” folder located at https://osf.io/a24dp/.
**Table S4.** Stimuli for the comprehension task (non‐semantic task).
**Table S5.** The locations of the activation peaks (MNI coordinates) from the GLM analyses.
**Table S6.** The locations of the activation peaks (MNI coordinates) from the ICA.
**Figure S1.** GLM results for the non‐semantic task. *T*‐maps were corrected for multiple comparisons using a voxel‐wise level significance threshold set at *p* < .001 with an FWE correction applied at the critical cluster level at *p* < .05.
**Figure S2.** GLM results for the semantic task. *T*‐maps were corrected for multiple comparisons using a voxel‐wise level significance threshold set at *p* < .001 with an FWE correction applied at the critical cluster level at *p* < .05.
**Figure S3.** GLM results for the semantic versus non‐semantic task. *T*‐maps were corrected for multiple comparisons using a voxel‐wise level significance threshold set at *p* < .001 with an FWE correction applied at the critical cluster level at *p* < .05.
**Figure S4.** Overlap between the *F*‐map relative to the conjunction analysis results (semantic and non‐semantic HC&LC vs. NC) and the *T*‐map reflecting the Left Executive Control Network (LECN) derived from the ICA. Both *F‐* and *T*‐maps were corrected for multiple comparisons using a voxel‐wise level significance threshold set at *p* < .001 with an FWE correction applied at the critical cluster level at *p* < .05.

## Data Availability

The data will be made available at http://www.mrc-cbu.cam.ac.uk/publications/opendata. The full list of stimuli of the non‐semantic task can be downloaded from https://osf.io/a24dp/.
